# BMC Medical Education reviewer acknowledgement 2014

**DOI:** 10.1186/s12909-015-0287-4

**Published:** 2015-03-18

**Authors:** Clare Partridge

**Affiliations:** BioMed Central, Floor 6, 236 Gray’s Inn Road, London, WC1X 8HB UK

## Abstract

The editors of BMC Medical Education would like to thank all our reviewers who have contributed to the journal in Volume 14 (2014).

**Aase Aamland**

Norway

**Saeed Abbasi**

Iran

**Hamza Abdulghani**

Saudi Arabia

**Joseph Acker**

Australia

**Michael Adams**

USA

**Omokhoa Adeleye**

Nigeria

**Rajesh Aggarwal**

Canada

**Ducksun Ahn**

South Korea

**Enrico Aititni**

Italy

**Rola Ajjawi**

UK

**Justine Aka**

UK

**Omer Akyol**

Turkey

**Ahmed Al Ansari**

Bahrain

**Fares Alahdab**

USA

**John Albarran**

UK

**Urs-Vito Albrecht**

Germany

**Sami Al-Dubai**

Malaysia

**Mohamed Al-Eraky**

Egypt

**Syeda Ali**

Pakistan

**Guillaume Alinier**

Qatar

**Abdulrahman Al-Khateeb**

Saudi Arabia

**Patricia Alpert**

USA

**Raja Alyusuf**

Bahrain

**Lucy Ambrose**

UK

**Cheryl Amundsen**

Canada

**Gowri Anandarajah**

USA

**Douglas Ander**

USA

**Allen Andrade**

USA

**Jane Andreasen**

Denmark

**Vuokko Anttonen**

Finland

**Thelma Leite De Araujo**

Brazil

**Elize Archer**

South Africa

**Afua Arhin**

USA

**Stephen Aronoff**

USA

**Parisa Aslani**

Australia

**Shervin Assari**

USA

**John Atherton**

Australia

**Trevor Austin**

UK

**Elaine Ayres**

USA

**Richard Ayres**

UK

**Samy Azer**

Malaysia

**Warwick Bagg**

New Zealand

**Walter Baile**

USA

**Paul Baker**

UK

**Chinthaka Balasooriya**

Australia

**Joan Ballantine**

UK

**Daniel Banks**

USA

**Farihan Barghouti**

Jordan

**Patrick Barlow**

USA

**Stephen Barnett**

Australia

**Anne Baroffio**

Switzerland

**Mark Barrow**

New Zealand

**Chris Beauchamp**

Canada

**Gary Beck**

USA

**Jochanan Benbassat**

Israel

**Deirdre Bennett**

Ireland

**Anne-Marie Bergh**

South Africa

**Alicia Bergman**

USA

**Andrea Bialocerkowski**

Australia

**Henning Biermann**

Germany

**Pierre Bigot**

France

**Federico Bilotta**

Italy

**Hudson Birden**

Australia

**Nettie Blankenstein**

Netherlands

**Julia Blitz South**

Africa

**Marcus Bloice**

Austria

**Robert Bloodgood**

USA

**Carrie Bohnert**

USA

**Harold Bok**

Netherlands

**Fiona Boland**

Ireland

**Valdes Bollela**

Brazil

**Andrew Bonney**

Australia

**Eveline Booij**

Netherlands

**Miles Bore**

Australia

**Henny Boshuizen**

Netherlands

**Hans Martin Bosse**

Germany

**Joseph Donald Boudreau**

Canada

**Judith Bowen**

USA

**Lesley Bowker**

UK

**Malcolm Boyle**

Australia

**Jadranka Bozikov**

Croatia

**Domingo Braile**

Brazil

**Sylvie Branchereau**

Germany

**Jan Breckwoldt**

Switzerland

**Susan Bridges**

China

**Guy Brock**

USA

**Kathleen Brooks**

USA

**George Brown**

UK

**Ardith Brunt**

USA

**Ryan Brydges**

Canada

**Sharon Buckley**

UK

**Irem Budakoglu**

Turkey

**Ishfaq Bukhari**

Saudi Arabia

**Bryan Burford**

UK

**Annette Burgess**

Australia

**Louise Burgoyne**

Ireland

**Hilary Burton**

UK

**Jamiu Busari**

Netherlands

**Andrew Butler**

USA

**Sandra Buttigieg**

Malta

**Petra Bywood**

Australia

**Carrie Cameron**

USA

**Craig Campbell**

Canada

**Ben Canny**

Australia

**Peter Cantillon**

Ireland

**Marian Carey**

UK

**Nicola Carey**

UK

**Heather Carnahan**

Canada

**Rosemary Caron**

USA

**Sandra Carr**

Australia

**Madeline Carter**

UK

**Irene Carvalho**

Portugal

**Marie Caulfield**

USA

**Afonso Cavaco**

Portugal

**Judith Cave**

UK

**Nora Celebi**

Germany

**Joao Cerqueira**

Portugal

**Bernard Cerutti**

Switzerland

**Thomas Chacko**

India

**Wy Chan**

UK

**Ming-Ka Chan**

Canada

**Philip Chan**

UK

**Rodger Charlton**

UK

**Aleda Chen**

USA

**Yen-Yuan Chen**

Taiwan

**Julie Chen**

Hong Kong

**Minhuey Chen**

Taiwan

**Chu Chiang**

UK

**John Chibnall**

USA

**Anna Chisholm**

UK

**Tzong-Shinn Chu**

Taiwan

**Jane Chueh**

USA

**Maria Chun**

USA

**Ching-Wa Chung**

UK

**Thomas Churchill**

Canada

**Sarah Clark**

USA

**Jennifer Cleland**

UK

**David Coady**

UK

**Fran Cogen**

USA

**Deborah Cohen**

UK

**Michael Cohn**

USA

**F Cohn**

USA

**James Colbert**

USA

**Colin Coles**

UK

**John Collins**

Canada

**Hoffie Conradie**

South Africa

**David Cook**

USA

**Siobhan Cooke**

UK

**Katherine S. Corker**

USA

**Martina Cornel**

Netherlands

**Selma Corovic**

Slovenia

**Melissa Cortez**

USA

**Ozlem Coskun**

Turkey

**Patrício Costa**

Portugal

**Ian Couper**

South Africa

**Molly Courtenay**

UK

**David Cox**

UK

**Walter Coyle**

USA

**Alisha Crayton**

UK

**Jim Crossley**

UK

**Richard Cruess**

Canada

**Sylvia Cruess**

Canada

**Rodney Crutcher**

Canada

**David Cumin**

New Zealand

**Daniel Cummings**

UK

**Vernon Curran**

Canada

**Liviana Da Dalt**

Italy

**Ana Da Silva**

UK

**Marie Dacey**

USA

**Jane Dacre**

UK

**Lalit Dandona**

India

**Kay Daniels**

USA

**Arianne Dantas**

Australia

**Deepak Dath**

Canada

**David Davies**

UK

**Margaretha Christine De Bruijne**

Netherlands

**Walter De Caro**

Italy

**Chris de Gara**

Canada

**Sandra De Groote**

USA

**Peter de Jong**

Netherlands

**Guillermo de la Calle**

Spain

**Clare Delany**

Australia

**Ahmet Demican**

Turkey

**Paul Denny**

New Zealand

**Isabelle Desjardins**

Canada

**Anouk Dev**

Australia

**Curtiland Deville**

USA

**Chris Dewberry**

UK

**Sanjiv Dhingra**

Canada

**Marie-Louise Dick**

Australia

**Peter Dieckmann**

Denmark

**Christina Dilaveri**

USA

**Helen Dimaras**

Canada

**Georgios Dimitrakakis**

UK

**Mark Dixon**

Ireland

**Patricia Dobkin**

Canada

**Eva Doherty**

Ireland

**D Dolmans**

Netherlands

**Melissa Dominicé Dao**

Switzerland

**Frank Domino**

USA

**Miltiadis Douvoyiannis**

Greece

**Jon Dowell**

UK

**Paul Drain**

USA

**Brian Drolet**

USA

**Jeff Druck**

USA

**Adam Dubrowski**

Canada

**Robert Dudas**

USA

**Eileen Duggan**

Ireland

**Roger Dunston**

Australia

**Samuel Edelbring**

Sweden

**Gudrun Edgren**

Sweden

**Carolyn Ehrlich**

Australia

**Solvig Ekblad**

Sweden

**Charles Eldridge**

USA

**Diann Eley**

Australia

**Bernice Elger**

UK

**Rodrigo Elizondo Omaña**

Mexico

**Suzan Elsayed**

USA

**Lars Enochsson**

Sweden

**Christabel Enweronu-Laryea**

Ghana

**Geurt Essers**

Netherlands

**Jorge Esteves**

UK

**Catrin Evans**

UK

**David Evans**

UK

**Phil Evans**

UK

**Goetz Fabry**

Germany

**Angela Fan**

Taiwan

**Li Felländer-Tsai**

Sweden

**Marc Felzen**

Germany

**Robert Ferguson**

USA

**Simon Field**

Canada

**Arnstein Finset**

Norway

**Evridiki Fioratou**

UK

**J.E.F. Fitzgerald**

UK

**Silvia Florescu**

Romania

**Cornelia Fluit**

Netherlands

**Henry Fomundam**

South Africa

**Alice Fornari**

USA

**Lewis Foxhall**

USA

**Jason Frank**

Canada

**Reiner Frank**

Germany

**Kristin Fraser**

Canada

**Adrian Freeman**

UK

**Jan C. Frich**

Norway

**Yun Fu**

China

**Abraham Fuks**

Canada

**Daniel Furmedge**

UK

**Robert Gagnon**

Canada

**Anthony Gallagher**

Ireland

**Rousseau Gama**

UK

**Ana Garces**

Guatemala

**Paul Garrud**

UK

**Gabor Gazdag**

Hungary

**James Gazzard**

UK

**Edward Gehringer**

USA

**Orsolya Genzel**

Germany

**Ahmad Gharehbaghian**

Iran

**Neil Gibson**

Canada

**Benjamin Gierk**

Germany

**Gerard Gill**

Australia

**Ronald Gimbel**

USA

**Daniel Girzadas Jr.**

USA

**Peter Gliatto**

USA

**Leanne Goding**

Australia

**Jeffrey Gold**

USA

**John Goldie**

UK

**Harumi Gomi**

Japan

**Morris Gordon**

UK

**Süleyman Görpelioglu**

Turkey

**Gertraud Gradl**

Germany

**Andrew Grant**

UK

**Maria Caterina Grassi**

Italy

**Andrew Gratrix**

UK

**Lionel Green-Thompson**

South Africa

**Ryan Greysen**

USA

**Barbara Griffin**

Australia

**Jan Groener**

Germany

**Doug Gruner**

Canada

**Larry Gruppen**

USA

**Kay Guccione**

UK

**Anthony Guerrero**

USA

**Simon Guild**

UK

**Lyn Gum**

Australia

**Freedom Nkhululeko Gumedze**

South Africa

**Murat Gunaydin**

Turkey

**Susan Guralnick**

USA

**Erol Gurpinar**

Turkey

**Ana Estela Haddad**

Brazil

**Carol Haigh**

UK

**Harry Haigler**

USA

**Ladonna S. Hale**

USA

**Samuel Hall**

UK

**John Hamilton**

Australia

**David I. Hanauer**

USA

**Trevor Hancock**

Canada

**Lezley-Anne Hanna**

UK

**Wolfgang Hannöver**

Germany

**Inam Haq**

UK

**Sigrid Harendza**

Germany

**Don Harting**

USA

**Daniel Hartung**

USA

**Wolfgang Hasemann**

Switzerland

**Johannes Hauswaldt**

Germany

**Shogo Hayashi**

Japan

**Osman Hayran**

Turkey

**Richard Hays**

Australia

**Iman Hegazi**

Australia

**Inga Hege**

Germany

**Kristin Heggen**

Norway

**Esther Helmich**

Netherlands

**Catherine Hennessy**

UK

**Gunnel Hensing**

Sweden

**Anne Herrmann-Werner**

Germany

**Hannah Hesselgreaves**

UK

**Sharon Hewner**

USA

**Tim Heywood**

UK

**Andrew Hill**

New Zealand

**Are Holen**

Norway

**Neil Holland**

USA

**Andreas Holzinger**

Austria

**Caroline Homer**

Australia

**Matt Homer**

UK

**Juliette Hommes**

Netherlands

**Michael Hortsch**

USA

**Eleanor Hothersall**

UK

**John Howard**

UK

**Chin-Chou Huang**

Taiwan

**Judith Hudson**

Australia

**Jannie Hugo**

South Africa

**Henk Huijser**

Australia

**Michael Hulme**

USA

**Gerald Humphris**

UK

**Christopher Huntley**

UK

**Shereen Hussein**

UK

**Sören Huwendiek**

Switzerland

**Jennifer Hyland**

UK

**Judith Ibison**

UK

**Carel Ijsselmuiden**

Switzerland

**December S.K. Ikah**

UK

**Dragan Ilic**

Australia

**Shehnaz Ilyas**

India

**Marita Rohr Inglehart**

USA

**Laili Irani**

USA

**Karin E Isaksson Ro**

Norway

**Peter Iserbyt**

Belgium

**Siddek Isreb**

UK

**Sarah Itam**

UK

**Alan Jaap**

UK

**Thomas Jaarsma**

Netherlands

**Christine Jackson**

UK

**Animesh Jain**

India

**Mohammad Jalili**

Iran

**Sharmila Jandial**

UK

**William Jeffries**

USA

**Kate Jennings**

USA

**Sarah Jeong**

Australia

**Anthony Jerant**

USA

**Viju John**

USA

**Carolyn Johnston**

UK

**Michael Jones**

Australia

**Rhys Jones**

New Zealand

**Terry Judd**

Australia

**Florian Junne**

Germany

**Noelle Junod Perron**

Switzerland

**John Kalbfleisch**

USA

**Susanne Kalén**

Sweden

**Elsbeth Kalenderian**

USA

**Summers Kalishman**

USA

**Jonathan Kam**

Australia

**Nazan Karaoglu**

Turkey

**Khalid Karim**

UK

**Randi Karlsen**

Norway

**Orit Karnieli-Miller**

Israel

**Paramjit Kaur**

India

**Argyro Kavadella**

Greece

**Iain Keenan**

UK

**Carolina Keijsers**

Netherlands

**Clare Kell**

UK

**Frances Kelly**

New Zealand

**Martina Kelly**

Canada

**Maureen E Kelly**

Ireland

**Robert Kennedy**

USA

**Jean Ker**

UK

**Jung-Hee Kim**

South Korea

**Kyong-Jee Kim**

South Korea

**Ian Kinchin**

UK

**Sharla King**

Canada

**Sue Kirsa**

Australia

**Marc Kiviniemi**

USA

**Niels Kristian Kjaer**

Denmark

**Zalika Klemenc-Ketis**

Slovenia

**Rhona Knight**

UK

**Richard Knox**

UK

**Ansgar Koechel**

Germany

**Jennifer Kogan**

USA

**Joseph Kolars**

USA

**Nobuyasu Komasawa**

Japan

**Lars Konge**

Denmark

**Andrzej Kononowicz**

Sweden

**Malcolm Koo**

Taiwan

**Jan Kooloos**

Netherlands

**Veronika Kopp**

Germany

**Ross Koppel**

USA

**Markus Krautter**

Germany

**Edward Krupat**

USA

**Sunčana Kukolja**

Croatia

**Kulamakan Kulasegaram**

Canada

**Erik Kulstad**

USA

**Arno Kumagai**

USA

**Viji Kurup**

USA

**Nanon Labrie**

Switzerland

**Naomi Lacy**

USA

**Nai Ming Lai**

Malaysia

**Anita Laidlaw**

UK

**Trevor Lambert**

UK

**Johanna Lammintakanen**

Finland

**Samsun Lampotang**

USA

**Sean Langenfeld**

USA

**Kaye Lasserre**

Australia

**Jan Lauter**

Germany

**Renee Lawrence**

USA

**Elizabeth Lazzara**

USA

**David Leaf**

USA

**Ming-Been Lee**

Taiwan

**Ronny Lehmann**

Germany

**Heidi Lempp**

UK

**Sum Leong**

Ireland

**Jimmie Leppink**

Netherlands

**Ruth Levine**

USA

**Linda Lewin**

USA

**Che-Wei Lin**

Taiwan

**Michelle Lincoln**

Australia

**Matthew Lineberry**

USA

**Annemiek Linn**

Netherlands

**Andrew Lockey**

UK

**Tai Lockspeiser**

USA

**Jennifer Loertscher**

USA

**Benjamin Lok**

USA

**Sten Ludvigsen**

Norway

**Bjarke Lund Sorensen**

Denmark

**Dale Lupu**

USA

**Gregory Lydall**

UK

**Joanne Lymn**

UK

**Nigel Mabvuure**

UK

**Alex Macario**

USA

**David Maclaren**

Australia

**Parker Magin**

Australia

**John Mahan**

USA

**Cornelia Mahler**

Germany

**Carlo Maley**

USA

**Stephen Malkoski**

USA

**Silvia Mamede**

Netherlands

**Katri Manninen**

Sweden

**Alvaro Margolis**

Uruguay

**Stephan Marsch**

Switzerland

**Ana Marusic**

Croatia

**Joanna Matthan**

UK

**Nikos Mattheos**

Hong Kong

**Brian Mavis**

USA

**Simon Maxwell**

UK

**Win May**

USA

**Liza McCann**

UK

**Elizabeth McClure**

USA

**Geoffrey McColl**

Australia

**Sindy McCrystle**

USA

**Katie McDonald**

USA

**Maureen McEvoy**

Australia

**Karen McKelvie**

UK

**Suzanne McKenzie**

Australia

**Danette McKinley**

USA

**John McLachlan**

UK

**Daniel McLaughlin**

UK

**Michelle McLean**

Australia

**Lucy McLellan**

UK

**Chris McManus**

UK

**Doug McMillan**

Canada

**Wendy McMillan**

South Africa

**H. Patrick McNeil**

Australia

**John McNulty**

USA

**Craig Mellis**

Australia

**Stewart Mennin**

USA

**Sultan Ayoub Meo**

Saudi Arabia

**Annette Mercer**

Australia

**Juanita Merchant**

USA

**M. David Merrill**

USA

**Aliapsha Meysamie**

Iran

**Misa Mi**

USA

**Felise Milan**

USA

**Lukas Mileder**

Austria

**David Miller**

USA

**Susan Miller**

Australia

**Marianna Milosavljevic**

Australia

**Sylvia Mione**

Belgium

**Mayank Kumar Mittal**

USA

**Andreas Möltner**

Germany

**Giovanni Moneta**

UK

**Koenraad Monsieurs**

Belgium

**Calvin Moorley**

UK

**Shabir Moosa**

South Africa

**Prue Morgan**

Australia

**Andrew Moriarty**

UK

**Christopher P. Morley**

USA

**Patricia Morley-Forster**

Canada

**Tracy Morrison**

Australia

**Aloysius Mubuuke**

Uganda

**Vivek Mudera**

UK

**Brigitte Mueller-Hilke**

Germany

**Azeem Muhammad Abdul**

Pakistan

**Patricia Mullan**

USA

**Don Munro**

Australia

**Tohru Murakami**

Japan

**Nick Murch**

UK

**Douglas Murphy**

UK

**Ahmad Mustafa**

UK

**Sun Jung Myung**

South Korea

**Alisa Nagler**

USA

**Mona Nasir**

UK

**Udayakumar Navaneethan**

USA

**Adam Nelson**

Australia

**Joan Nelson**

USA

**Mathieu Nendaz**

Switzerland

**Danielle Ni Chroinin**

Australia

**Sandra Nicholson**

UK

**Manuel Nienkemper**

Germany

**Christoph Nikendei**

Germany

**Andreas Nikolis**

Canada

**Somashekhar Nimbalkar**

India

**Annie Noble**

UK

**Thomas E. Norris**

USA

**Zineb Nouns**

Switzerland

**Bridget Obrien**

USA

**Robert O'Brien**

Australia

**Hugh O'Connor**

Ireland

**Sabine Oertelt-Prigione**

Germany

**Antoinette Ofili**

Nigeria

**Curtis Olson**

USA

**Mariann Olsson**

Sweden

**Peter O'Meara**

Australia

**Christopher O'Neal**

USA

**Anna O'Neill**

UK

**Hirotaka Onishi**

Japan

**Jay Orlander**

USA

**Kazeem Oshikoya**

Nigeria

**Abiodun Osiyemi**

Nigeria

**Jan Östergren**

Sweden

**Anthony O'Sullivan**

Australia

**Anna Oswald**

Canada

**Gozde Ozakinci**

UK

**Nilgun Ozcakar**

Turkey

**John Paige**

USA

**Alvisa Palese**

Italy

**Edward Palmer**

Australia

**Harshal Pandve**

India

**Andrew Papanikitas**

UK

**Klara Papp**

USA

**Elaine Papps**

New Zealand

**Malcolm Parker**

Australia

**Dean Parmelee**

USA

**Rona Patey**

UK

**Catherine Paton**

UK

**Leslie Payne**

USA

**Godfrey Pell**

UK

**Jaixi Peng**

China

**Denis Pereira Gray**

UK

**Colin Perry**

UK

**Susan Persky**

USA

**John Peteet**

USA

**Ray Peterson**

Australia

**Ingrid Philibert**

USA

**Victoria Phillips**

USA

**Jobeth Pilcher**

USA

**Tahir Pillay**

South Africa

**Florian Pilz**

Germany

**Sandro Pinheiro**

USA

**Kim Piper**

UK

**Sabrina Pit**

Australia

**Karen Plager**

USA

**Katherine C Pollard**

UK

**Phillippa Poole**

New Zealand

**Giampiero Porzio**

Italy

**Kevin Pottie**

Canada

**David Alan Powis**

Australia

**Gill Price**

UK

**Shane Pritchard**

Australia

**Ian Puddey**

Australia

**Sue Pullon**

New Zealand

**Mohammad Qadire**

Jordan

**Magdalena Zuzanna Raban**

Australia

**Athanasios Raikos**

Australia

**Charles Raison**

USA

**Linda Raphael**

USA

**Savithiri Ratnapalan**

Canada

**Tobias Raupach**

Germany

**Mark Raymond**

USA

**Anne-Marie Reid**

UK

**Luke Reid**

UK

**Stephen Reid**

South Africa

**Marcel Reinders**

Netherlands

**Gary Reisfield**

USA

**Jan-Joost Rethans**

Netherlands

**Jayne Reuben**

USA

**Arnoldo Riquelme**

Chile

**Torsten Risor**

Norway

**Donald Risucci**

USA

**William Roberts**

USA

**Christopher Roberts**

Australia

**Martin Roberts**

UK

**José Rodríguez**

USA

**Susan Roelofs**

Canada

**Sue Roff**

UK

**David Rogers**

USA

**Anke Rohwer**

South Africa

**Martina Rojnic Kuzman**

Croatia

**Damian Roland**

UK

**Marco Roos**

Germany

**Gail Rose**

USA

**Steven Rose**

USA

**Glenn Rosenbluth**

USA

**Mike Rowson**

UK

**Mariah Rudd**

USA

**Joy Rudland**

New Zealand

**Roger Ruiz Moral**

Spain

**Homayoun Sadeghi-Bazargani**

Iran

**Hazim Sadideen**

UK

**Elianna Saidenberg**

Canada

**Tetsuro Sakai**

USA

**Abdus Salam**

Malaysia

**Helen Salisbury**

UK

**Murali Sambasivan**

Malaysia

**Dario Sambunjak**

Croatia

**Sally Santen**

USA

**Emmanuel Sanya**

Nigeria

**Joan Sargeant**

Canada

**Kamran Sattar**

Saudi Arabia

**Adam Sawatsky**

USA

**Marina Sawdon**

UK

**Samantha Scallan**

UK

**Stefan K. Schauber**

Germany

**Christian Scheffer**

Germany

**Joerg Schelling**

Germany

**Sanneke Schepman**

Netherlands

**Albert Scherpbier**

Netherlands

**Marcus Schiltenwolf**

Germany

**Ralf Schmidmaier**

Germany

**Thomas Schmidt**

USA

**Kai Philipp Schnabel**

Switzerland

**Francine Schneider**

Netherlands

**Ellie Schoenbaum**

USA

**Deborah Joy Schofield**

Australia

**Ingrid Scholten**

Australia

**Erik Schoon**

Netherlands

**Michael Schreiber**

Germany

**Andreas Schröder**

Denmark

**David Schulman**

USA

**Jobst-Hendrik Schultz**

Germany

**Daniel Schumacher**

USA

**Richard Schuster**

USA

**Katrin Schüttpelz-Brauns**

Germany

**Todd Schwartz**

USA

**Thomas Schwenk**

USA

**Dean Seehusen**

USA

**Doris Selvaratnam**

Malaysia

**Tarun Sen Gupta**

Australia

**Maria Helena Senger**

Brazil

**Walter Sermeus**

Belgium

**Turan Set**

Turkey

**Tanmeet Sethi**

USA

**Milton Severo**

Portugal

**Patricia Sexton**

USA

**Raluca Sfetcu**

Romania

**Sami Shaban**

United Arab Emirates

**Nachiket Shankar**

India

**E Shen**

USA

**Renslow Sherer**

USA

**Wesley Shrum**

USA

**Boaz Shulruf**

Australia

**Matthew Sibbald**

Canada

**Melanie Simon**

Germany

**Veena Singaram**

South Africa

**Helge Skirbekk**

Norway

**David Sleet**

USA

**Arlene Smaldone**

USA

**Claire Smith**

UK

**Tony Smith**

Australia

**Sarah Smithson**

UK

**David Snadden**

Canada

**Jonathan Snashall**

USA

**Suzanne Snodgrass**

Australia

**Dejano T. Sobral**

Brazil

**Diantha Soemantri**

Indonesia

**Marc Soethout**

Netherlands

**John Sokolowski**

USA

**Tore Sorlie**

Norway

**Gina Sorrentino**

USA

**Jeannette South-Paul**

USA

**Janet Specht**

USA

**Judith Spiers**

Canada

**Dhiraj Srivastava**

India

**Renee Stalmeijer**

Netherlands

**Keith Steele**

UK

**Karen Stegers-Jager**

Netherlands

**Beat Steiner**

USA

**Kirsten Steinhausen**

Germany

**Fred Stevens**

Netherlands

**Michael Stevenson**

UK

**Philipp Stieger**

Germany

**Jan Stiepak**

Germany

**Heather Stoffel**

USA

**Mark Sujan**

UK

**Annelie Sundler Johansson**

Sweden

**Tarja Suominen**

Finland

**Krishna Mohan Surapaneni**

India

**Sadasivam Suresh**

Australia

**Meenakshi Swamy**

UK

**Waleed Sweileh**

Palestinian Territory

**Jamsheer Talati**

Pakistan

**Jo-Ann Talbot**

Canada

**Vicky Tallentire**

Australia

**Amy Tan**

Canada

**Howard Tandeter**

Israel

**Marie Tarrant**

Hong Kong

**Murat Tavlasoglu**

Turkey

**Bineyam Taye**

Ethiopia

**Benjamin Taylor**

USA

**Olle Ten Cate**

Netherlands

**Naichia Teng**

Taiwan

**Cheong Lieng Teng**

Malaysia

**Pim Teunissen**

Netherlands

**Rosalie Thackrah**

Australia

**Axel Themmen**

Netherlands

**David Therriault**

USA

**Ravi Thiagarajan**

USA

**Charles R. Thomas**

USA

**Bart Thoonen**

Netherlands

**Sean Tierney**

Ireland

**Paul Tiffin**

UK

**David Tiller**

Australia

**Julie K. Tilson**

USA

**Naobumi Tochigi**

Japan

**Jonathon Tomlinson**

UK

**William Tormey**

Ireland

**Jane Torrie**

New Zealand

**Franziska Trede**

Australia

**John Triano**

Canada

**Konstantinos Triantafyllou**

Greece

**Shih-Li Tsai**

Taiwan

**Ellen Tullo**

UK

**Feng-Cheng Tung**

Taiwan

**Janis Tupesis**

USA

**Mike Tweed**

New Zealand

**Reidar Tyssen**

Norway

**Sebastian Uijtdehaage**

USA

**Robin Urquhart**

Canada

**Jinan Usta**

Lebanon

**Maritta Välimäki**

Finland

**Mauro Vaccarezza**

Italy

**Thea van de Mortel**

Australia

**John van den Dobbelsteen**

Netherlands

**Cees van der Vleuten**

Netherlands

**Johannes C. van der Wouden**

Netherlands

**Jouke van der Zee**

Netherlands

**Nynke van Dijk**

Netherlands

**Philip van Eijndhoven**

Netherlands

**Daniel van Hoving**

South Africa

**Thed van Leeuwen**

Netherlands

**Dirk van Rooy**

Australia

**Susan van Schalkwyk**

South Africa

**Marta van Zanten**

USA

**Tom van Zundert**

Belgium

**Prathibha Varkey**

USA

**Sonia Vasconcelos**

Brazil

**Gary Velan**

Australia

**Yoga Nathan Velupillai**

Ireland

**Arun Venkatesan**

USA

**Petra Verdonk**

Netherlands

**Pierre Verger**

France

**Danielle Verstegen**

Netherlands

**Shlomo Vinker**

Israel

**Claudio Violato**

Canada

**Silvia Visentin**

Italy

**Ana Vujaklija Brajkovic**

Croatia

**Rashmi Vyas**

India

**Rukhsana W Zuberi**

Pakistan

**Jean Wallace**

Canada

**Catharine Walsh**

Canada

**Allyn Walsh**

Canada

**Helena Ward**

Australia

**Simon Watmough**

UK

**Roger Watson**

UK

**Andy Wearn**

New Zealand

**Alexandra Webb**

Australia

**Gillian Webb**

Australia

**Craig Webster**

New Zealand

**Jennifer Mary Weller**

New Zealand

**Leana Wen**

USA

**Marjorie Wenrich**

USA

**Colin West**

USA

**Olwyn Westwood**

UK

**Shari Whicker**

USA

**Franklin White**

Canada

**Sue Whittle**

UK

**Gerald Wibbecke**

Germany

**Carilyn Wieland**

USA

**Sharon Wiener-Ogilvie**

UK

**Marjo Wijnen-Meijer**

Netherlands

**Ingrid Willaing**

Denmark

**Simon Willcock**

Australia

**Laura Willett**

USA

**Brett Williams**

Australia

**Brent Williams**

USA

**Francie Williams**

USA

**Gregory Williams**

New Zealand

**Jude Williams**

Australia

**Cathy Williamson**

UK

**Graham Richard Williamson**

UK

**Hamish Wilson**

New Zealand

**Ian Wilson**

Australia

**Adam Wilson**

USA

**Donna Windish**

USA

**Peter Winocour**

UK

**Ulrich Woermann**

Switzerland

**Traci Wolbrink**

USA

**Stanley Wong**

USA

**Yut Lin Wong**

Malaysia

**Michael Woo**

Canada

**Anouk Wouters**

Netherlands

**David Wright**

UK

**Scott Wright**

USA

**Mary Wroblewski**

USA

**Peter Wyer**

USA

**Theodoros Xanthos**

Greece

**Shifu Xiao**

China

**Siyan Xu**

USA

**John Yaphe**

Portugal

**Ahmed Yaqinuddin**

Pakistan

**Sarah Yardley**

UK

**Janet Yates**

UK

**Peter Yeates**

UK

**Liuhua Ying**

China

**Tzu-Chieh Yu**

New Zealand

**Carole Yue**

USA

**Zamros Yusof**

Malaysia

**Zareen Zaidi**

Pakistan

**Luiz Carlos Zeferino**

Brazil

**Elma Zoboli**

Brazil

**Michaela Zupanic**

Germany

